# [Retracted] Crocin protects against dexamethasone-induced osteoblast apoptosis by inhibiting the ROS/Ca^2+^-mediated mitochondrial pathway

**DOI:** 10.3892/mmr.2025.13561

**Published:** 2025-05-12

**Authors:** Zhigang Nie, Shuang Deng, Lei Zhang, Sen Chen, Qiang Lu, Hao Peng

Mol Med Rep 20: 401–408, 2019; DOI: 10.3892/mmr.2019.10267

Following the publication of the above paper, it was drawn to the Editor's attention by a concerned reader that, regarding the flow cytometric plots shown in Fig. 2A on p. 404, the ‘Ctrl’ and ‘Dex + Cro 5μM’ panels appeared to share a very similar distribution of data in their lower left quadrants, whereas the other parts of the panels appeared differently, which would not have been anticipated if these experiments had been performed discretely under different experimental conditions, suggesting a fundamental flaw either in the way in which these experiments were performed or in how the results were outputted.

The Editor of *Molecular Medicine Reports* has decided that this paper should be retracted from the Journal on account of a lack of confidence in the presented data. The authors were asked for an explanation to account for these concerns, but the Editorial Office did not receive a reply. The Editor apologizes to the readership for any inconvenience caused.

